# Supplementary material for: An applicable and efficient retrograde monosynaptic circuit mapping tool for larval zebrafish
Source: eLife. 2026 Sep 18;13:RP100880. doi: 10.7554/eLife.100880 (PMC13588618; doi:10.7554/eLife.100880)
Supplement: Peer review history

# Editor report: eLife Assessment

**DOI:** 10.7554/eLife.100880.4.sa0

**Authors:** Xin Duan

**Affiliations:** University of California, San Francisco United States

**Keywords:** Compelling, Important

This **important** technical development for neural circuit tracing in larval zebrafish consists in an enhanced rabies virus for improved retrograde transneuronal tracing, supporting a new method for combined structural and functional brain mapping which is demonstrated with **compelling** evidence. The work will interest zebrafish neurobiologists for the identification of neuronal connectivity patterns while simultaneously monitoring circuit activity.

# Referee report: Reviewer #2 (Public review)

**DOI:** 10.7554/eLife.100880.4.sa1

The study by Chen, Deng et al. aims to develop an efficient viral transneuronal tracing method that enables retrograde tracing in larval zebrafish. The authors utilize pseudotyped rabies virus that can be targeted to specific cell types using the EnvA-TvA system.

Pseudotyped rabies virus has been used extensively in rodent models and, in recent years, has begun to be developed for use in adult zebrafish. However, compared to rodents, the efficiency of spread in adult zebrafish is very low (~one upstream neuron labeled per starter cell). Additionally, there is limited evidence of retrograde tracing with pseudotyped rabies in the larval stage, which is when most functional neural imaging studies are conducted in the field. In this study, the authors systematically optimized several parameters for rabies tracing, including rabies virus strains, glycoprotein types, temperatures, expression construct designs, and the elimination of glial labeling. The optimal configurations developed by the authors are up to 5-10-fold higher than more commonly used configurations.

The results are compelling and support the conclusions.

# Author comment: Author response

**DOI:** 10.7554/eLife.100880.4.sa2

**Authors:** Tianlun Chen, Qiusui Deng, Kunzhang Lin, Xiudan Zheng, Xin Wang, Yongwei Zhong, Xinyu Ning, Ying Li, Fuqiang Xu, Jiulin Du, Xufei Du

**Affiliations:** Institute of Neuroscience, Key Laboratory of Brain Cognition and Brain-Inspired Intelligence Technology, Center for Excellence in Brain Science and Intelligence Technology, Chinese Academy of Sciences Shanghai China; https://ror.org/05qbk4x57 University of Chinese Academy of Sciences Beijing China; Institute of Neuroscience, Key Laboratory of Brain Cognition and Brain-Inspired Intelligence Technology, Center for Excellence in Brain Science and Intelligence Technology, Chinese Academy of Sciences Shanghai China; https://ror.org/05qbk4x57 University of Chinese Academy of Sciences Beijing China; Shenzhen Key Laboratory of Viral Vectors for Biomedicine, Shenzhen-Hong Kong Institute of Brain Science, Shenzhen Institutes of Advanced Technology, Chinese Academy of Sciences Shenzhen China; Institute of Neuroscience, Key Laboratory of Brain Cognition and Brain-Inspired Intelligence Technology, Center for Excellence in Brain Science and Intelligence Technology, Chinese Academy of Sciences Shanghai China; Institute of Neuroscience, Key Laboratory of Brain Cognition and Brain-Inspired Intelligence Technology, Center for Excellence in Brain Science and Intelligence Technology, Chinese Academy of Sciences Shanghai China; https://ror.org/05qbk4x57 University of Chinese Academy of Sciences Beijing China; Institute of Neuroscience, Key Laboratory of Brain Cognition and Brain-Inspired Intelligence Technology, Center for Excellence in Brain Science and Intelligence Technology, Chinese Academy of Sciences Shanghai China; Institute of Neuroscience, Key Laboratory of Brain Cognition and Brain-Inspired Intelligence Technology, Center for Excellence in Brain Science and Intelligence Technology, Chinese Academy of Sciences Shanghai China; Institute of Neuroscience, Key Laboratory of Brain Cognition and Brain-Inspired Intelligence Technology, Center for Excellence in Brain Science and Intelligence Technology, Chinese Academy of Sciences Shanghai China; Shenzhen Key Laboratory of Viral Vectors for Biomedicine, Shenzhen-Hong Kong Institute of Brain Science, Shenzhen Institutes of Advanced Technology, Chinese Academy of Sciences Shenzhen China; Institute of Neuroscience, Key Laboratory of Brain Cognition and Brain-Inspired Intelligence Technology, Center for Excellence in Brain Science and Intelligence Technology, Chinese Academy of Sciences Shanghai China; https://ror.org/05qbk4x57 University of Chinese Academy of Sciences Beijing China; https://ror.org/030bhh786 School of Life Science and Technology, ShanghaiTech University Shanghai China; Institute of Neuroscience, Key Laboratory of Brain Cognition and Brain-Inspired Intelligence Technology, Center for Excellence in Brain Science and Intelligence Technology, Chinese Academy of Sciences Shanghai China; https://ror.org/05qbk4x57 University of Chinese Academy of Sciences Beijing China

The following is the authors’ response to the previous reviews

**Reviewer #1 (Public review):**

(1) Presentation of Figures in the Response Letter

I would like to note that the figures included in the response letter would benefit from improved organization. For example, Author response image 1 lacks clarity for experimental conditions. From the response letter, my understanding is that a "Labeling rate index", Rg−Rn, was calculated to represent the difference in the rate of increase in labeling between neurons and glial across two time intervals based on experiments shown in Figure 2-figure supplement 1C and G. It seems that a mean convergence index was calculated for each experimental condition at each time point for glial and neurons, and then the differences in mean convergence index increase between time intervals were calculated for glial and neurons. The legend needs more detail to enhance clarity.

Yes, the “labeling rate index” (Rg−Rn) corresponds exactly to the reviewer’s understanding. Specifically, it quantifies the difference between neurons and glia in the increase of the mean convergence index across two defined time intervals, calculated separately for each experimental condition based on the experiments shown in Figure 2–figure supplement 1C and G.

To improve clarity, we have substantially revised the figure legend to explicitly describe (i) the definition of labeling rate, (ii) how the mean convergence index was computed for neurons and glia at each time point, (iii) how changes across time intervals were derived, and (iv) how to calculate the labeling rate index. In addition, we have moved this analysis to Figure 2-figure supplement 2 and cited it in Line 191.

Furthermore, the manuscript should clearly distinguish between figures generated from re-analysis of existing data and those based on newly conducted experiments. This distinction should be explicitly stated in the figure legends and/or main text.

I recommend that all response figures containing data integral to the authors' rebuttal be properly integrated into the manuscript's existing supplementary figure set, rather than remaining isolated in the response document. This would enhance clarity and ensure that key supporting data are fully accessible to readers. For instance, Author response image 1 can be integrated with Figure 2-figure supplement.

We appreciate the reviewers’ valuable suggestions. We have revised the figure legends and/or corresponding main text to clearly distinguish figures derived from re-analysis of existing data from those based on newly conducted experiments. In addition, all response figures containing data integral to our rebuttal have now been integrated into the current manuscript’s supplementary figure set.

Specifically, Author response images 1 and 3 have been incorporated into Figure 2–figure supplement 2 and Figure 2–figure supplement 3, respectively; Author response image 2 has been incorporated into Figure 1–figure supplement 2. Author response image 4 has been incorporated into Figure 1,2–figure supplement 1. These changes improve clarity and ensure that all supporting data are readily accessible to readers.

(2) Glial Cell Labeling and Specificity of Trans-Synaptic Spread

The authors provided a comprehensive and well-reasoned response to the concern regarding the labeling of radial glial cells. The inclusion of a dedicated section in the revised Discussion and response figures (possibly to be integrated with supplementary figures), strengthens the manuscript.

The authors have made an interesting observation in Author response image 2 that glial labeling was frequently observed near the soma and dendrites of starter cells, suggesting that transneuronal labeled glial cells may be synaptically associated with the starter neurons. Also astroglia starter cells lead to infection of nearby TVA-negative astroglia, suggesting astroglia-to- astroglia transmission.

I find the response scientifically satisfactory and appreciate the authors' transparency in addressing the limitations of their approach.

We thank the reviewer for the positive and thoughtful evaluation. As suggested, we have integrated the revised Discussion and the corresponding response figures into the main text and the supplementary figure set, ensuring that these observations and their interpretation are clearly presented and readily accessible to readers.

(3) Temperature Effects and Larval Viability

The authors' justification for raising larvae at 36C to improve labeling efficiency is reasonable. The supporting data indicating minimal impact on larval viability within the experimental timeframe are convincing. Referencing prior behavioral studies and including survival data under controlled conditions adds credibility to their claims. I find this issue satisfactorily addressed.

We thank the reviewer for this positive and constructive evaluation.

(4) Viral Toxicity and Dosage Considerations, Secondary Starter Cells

The authors present a well-reasoned explanation that viral cytotoxicity is primarily driven by replication and not by viral titer or injection volume. However, the inclusion of experimental data directly testing the effects of higher titer or volume on starter cell viability would have strengthened this point, particularly since such tests are relatively straightforward to perform.

We agree with the reviewer that directly testing the effects of viral titer and injection volume on starter cell viability would further strengthen this point. In practice, we have already used the highest CVS virus titer that could be reliably generated in our system. Therefore, we tested injection volumes of up to 20 nl and observed no detectable effect on starter cell survival, whereas higher injection volumes resulted in deformation of the larval brain, precluding their use.

Although not shown as a separate figure, these data informed our interpretation of viral toxicity, which is now described more clearly in the revised Discussion. We hope that this explanation and the clarified discussion adequately address the reviewer’s concern.

Regarding the potential contribution of secondary starter cells, the authors provide a convincing rationale for why such effects are unlikely under their sparse labeling conditions. However, in cases where TVA and G are broadly expressed-such as under the vglut2a promoter, as shown in Author response image 2 it would be valuable to directly evaluate this possibility experimentally. While the authors' interpretation is reasonable, empirical validation would further strengthen their conclusions.

We appreciate the reviewer’s interest in experimentally evaluating the potential contribution of secondary starter cells under conditions of broad TVA and G expression. In response, we performed additional viral tracing experiments in which TVA and G were driven by the excitatory neuronal marker *vglut2a* to achieve broad helper expression.

As shown in a representative case (Author response image 1), newly appearing tdTomato^+^ neurons were observed at the later time (6 vs 3 dpi, circles), many of which were spatially separated from EGFP^+^/tdTomato^+^ starter neurons identified at the early time point (3 dpi, dashed circles). Notably, a subset of these newly labeled tdTomato^+^ neurons colocalized with EGFP (6 vs 3 dpi, dashed cyan circles). These new EGFP^+^/tdTomato^+^ neurons may represent secondary starter cells or delayed infection of initially targeted starters. Interpretation of tdTomato^+^-only neurons (6 dpi, gray circles) is further complicated by variability in projection distance and synaptic strength, as short-range secondary-order (or multi-level) inputs and long-range first-order inputs may be labeled within similar time windows. In addition, in the presence of multiple primary or secondary starter neurons, unambiguous assignment of labeled inputs to specific starters remains challenging, even with high-temporal-resolution imaging.

Owing to these constraints, empirical identification of secondary (or multi-level) connections is not readily achievable with the current tracing strategy. A potential solution would be to combine pan-neuronal helper expression with spatiotemporally controlled activation, for example, through a transgenic line enabling light-inducible helper expression (e.g., G protein). Such an approach would enable delayed and cell-specific initiation of secondary (or multi-level) starters, thereby temporally separating long-range first-order inputs from multi-step circuit propagation and permitting input tracing of targeted cells, ultimately improving the spatiotemporal resolution of circuit mapping.

We have incorporated a dedicated section in the revised Discussion to clarify the applicable scenarios, limitations, and future directions of this viral tracing strategy in zebrafish.

**Author response image 1.** Recombinant RV-based viral tracing under broad helper expression conditions. Time-lapse (3 and 6 dpi) confocal images of the larval hindbrain showing recombinant RV-based viral tracing under broad helper expression (TVA and G, green) via *vglut2a* promoter-driven *UGNT*, following posterior hindbrain infection with CVS*d*G-tdTomato[EnvA] (magenta). Dashed circles, areas enriched with EGFP^+^/tdTomato^+^ neurons; gray circles, areas enriched with tdTomato^+^-only neurons; dashed white lines, hindbrain boundaries. C, caudal; R, rostral. Scale bars, 20 μm.

**Reviewer #2 (Public review):**

The study by Chen, Deng et al. aims to develop an efficient viral transneuronal tracing method that allows efficient retrograde tracing in the larval zebrafish. The authors utilize pseudotyped-rabies virus that can be targeted to specific cell types using the EnvA-TvA systems. Pseudotyped rabies virus has been used extensively in rodent models and, in recent years, has begun to be developed for use in adult zebrafish. However, compared to rodents, the efficiency of spread in adult zebrafish is very low (~one upstream neuron labeled per starter cell). Additionally, there is limited evidence of retrograde tracing with pseudotyped rabies in the larval stage, which is the stage when most functional neural imaging studies are done in the field. In this study, the authors systematically optimized several parameters of rabies tracing, including different rabies virus strains, glycoprotein types, temperatures, expression construct designs, and elimination of glial labeling. The optimal configurations developed by the authors are up to 5-10 fold higher than more typically used configurations.

The results are convincing and support the conclusions. There are some additional changes that are recommended:

(1) The new data included in the response to reviewer's letter are important to support the main conclusions and should be included in the manuscript.

We agree with the reviewer that the new data provided in the response are important for supporting the main conclusions. Accordingly, we have now incorporated all four figures from the response into the supplementary figure set of the revised manuscript and added the corresponding descriptions and discussion to the main text where appropriate.

(2) Line 357-362: This section should include all of the response letter figures and associated details. Additionally, the Author response image 3 is at odds with Fig 2-supplement 1G. In Author response image 3, ~75% of glial cells labeled at 4 dpi loses their fluorescence by 10 dpi. However, Figure 2-supplement 1G shows that glial overall labeling increases ~2 fold from 4 dpi to 10 dpi. This would suggest that the de novo labeling rate for glia is much higher than the net labeling rate calculated from the convergence index. The authors should clarify these findings.

We agree with the reviewer that the original section at Lines 357-362 should cite the relevant figures and include the associated details. We have now relocated this content to the Results section and incorporated the corresponding figures and descriptions.

In addition, we fully agree with the reviewer’s interpretation regarding the apparent discrepancy between the high loss rate of early-labeled glial cells (previously Author response image 3, now Figure 2—figure supplement 3) and the net increase in total glial labeling (Figure 2—figure supplement 1G). This pattern indicates that the net convergence index underestimates the true rate of de novo glial infection, as early labeled glial cells progressively lose detectable fluorescence while overall glial labeling continues to increase, implying ongoing de novo infection events outpace this loss. We have clarified this point in the Results section.

**Recommendations for the authors:**

**Reviewer #2 (Recommendations for the authors):**

The new data included in the response to reviewer letter are important to support the main conclusions and should be included in the manuscript.

This recommendation echoes the point raised in Reviewer #2’s Public Comment #1. As detailed in our response there, all new data originally included in the response letter have now been fully integrated into the manuscript’s supplementary figure set, with corresponding descriptions added to the main text.

Line 357-362: This section should include all of the Author response images and associated details. Additionally, Author response image 3 is at odds with Fig 2-supplement 1G. In Author response image 3, ~75% of glial cells labeled at 4 dpi loses their fluorescence by 10 dpi. However, Figure 2-supplement 1G shows that glial overall labeling increases ~2 fold from 4 dpi to 10 dpi. This would suggest that the de novo labeling rate for glia is much higher than the net labeling rate calculated from the convergence index. The authors should clarify these findings.

This recommendation echoes the concern raised in Reviewer #2’s Public Comment #2 regarding the apparent discrepancy between glial cell loss and the net increase in glial labeling. Please refer to our response to that comment for a detailed explanation. Briefly, we clarify that the continued increase in overall glial labeling despite substantial loss of early-labeled glia indicates a high rate of ongoing de novo infection that is not captured by net convergence index measurements alone. The relevant figure and associated details, including this clarification, have now been incorporated into the revised main text.

Data and description for response letter Figure 4 should be quantified and added to the manuscript.

Across nine infected larvae examined, initial infection was consistently restricted to TVA-positive astroglia, typically involving a single starter glial cell per larva. No viral spread was observed in three larvae injected with SAD*d*G-mCherry[EnvA], whereas astroglia-to-astroglia transmission was detected in three of six larvae injected with CVS*d*G-tdTomato[EnvA]. Importantly, no neuronal labeling was observed in any of the experiments. These quantitative data and descriptions, originally presented as Author response image 4, have now been incorporated into the main text as (Figure 1,2–figure supplement 1).
